# Comparison of N-Terminal Pro-B-Type Natriuretic Peptide Between Cats with Cardiogenic Arterial Thromboembolism and Cats with Occult Cardiomyopathy Without Arterial Thromboembolism

**DOI:** 10.3390/ani16020157

**Published:** 2026-01-06

**Authors:** Michelle A. Oranges, Lisa M. Freeman, Elizabeth A. Rozanski, Emily T. Karlin, John E. Rush

**Affiliations:** Department of Clinical Sciences, Cummings School of Veterinary Medicine, Tufts University, 200 Westboro Road, North Grafton, MA 01536, USA; michelle.oranges@tufts.edu (M.A.O.);

**Keywords:** arterial thromboembolism, biomarker, cardiomyopathy, congestive heart failure, echocardiography, NT-proBNP

## Abstract

To help assess the relationship between N-terminal pro-B-type natriuretic peptide (NT-proBNP) and feline cardiogenic arterial thromboembolism (ATE), the objective of this retrospective study was to compare plasma NT-proBNP concentrations between cats with cardiomyopathy that developed ATE (ATE group) and cats with occult cardiomyopathy that did not develop ATE within 1 year of testing (occult cardiomyopathy [OCM] group). Cats with cardiomyopathy and congestive heart failure (CHF) but no ATE (CHF group) were included for comparison. Cats with cardiomyopathy that had NT-proBNP testing were classified into ATE, OCM, or CHF groups. Clinical, echocardiographic, treatment, and NT-proBNP data from medical records were reviewed and compared among groups. A receiver operating characteristic curve was generated to develop a cutoff point for NT-proBNP. Cats were then compared based on this cutoff point. The ATE group (*n* = 25) had significantly higher NT-proBNP concentrations than the OCM group (*n* = 31; *p* < 0.001); there was no significant difference in NT-proBNP concentrations between the ATE and CHF groups (*p* = 0.92). The estimated optimal NT-proBNP cutoff point to separate OCM and ATE groups was 491 pmol/L (sensitivity = 96.0%, specificity = 93.5%). Cats with NT-proBNP > 491 pmol/L had a larger left atrium, thicker left ventricle, lower fractional shortening, and higher prevalence of spontaneous echogenic contrast and left atrial thrombi on echocardiography. These preliminary, hypothesis-generating findings suggest that NT-proBNP concentrations > 491 pmol/L may help detect cats with OCM at risk for ATE, but given the limitations of this retrospective study, prospective studies are needed to evaluate the potential utility of this measurement.

## Simple Summary

Arterial thromboembolism (ATE) or clot is a common and devastating complication of heart disease in cats. To help assess the relationship between the cardiac biomarker, N-terminal pro-B-type natriuretic peptide (NT-proBNP), and ATE, we retrospectively compared plasma NT-proBNP levels among cats with cardiomyopathy that developed ATE (ATE group), cats with cardiomyopathy that did not develop ATE within 1 year of testing (occult cardiomyopathy [OCM] group), and cats with cardiomyopathy and congestive heart failure (CHF) but no ATE (CHF group). Cats in the ATE group (*n* = 25) had significantly higher NT-proBNP levels than the OCM group but there was no significant difference in NT-proBNP concentrations between the ATE and CHF groups. A cutoff point for NT-proBNP that separated the ATE and OCM groups was determined to be 491 pmol/L. Cats with NT-proBNP > 491 pmol/L had a larger left atrium, thicker left ventricle, lower heart contractility, and were more likely to have signs suggesting a higher risk for ATE on ultrasound of the heart. These preliminary, hypothesis-generating findings suggest that NT-proBNP concentrations > 491 pmol/L may help detect cats with OCM at risk for ATE, but given the limitations of this retrospective study, prospective studies are needed to evaluate the potential utility of this measurement.

## 1. Introduction

Cardiogenic arterial thromboembolism (ATE) is a devastating complication of feline cardiomyopathy, affecting 5–28% of cats with hypertrophic cardiomyopathy (HCM) [1,2,3,4,5]. Cats with cardiomyopathy that develop ATE have significantly shorter survival times compared to cats with occult cardiomyopathy or congestive heart failure (CHF) without ATE [1,2,6]. Median survival times for cats not euthanized at the time of ATE diagnosis range from 2 to 12 months [1,2,7,8,9]. Anecdotally, the peracute presentation of feline ATE is distressing for owners, and there is a tendency for veterinarians to view feline ATE as a fatal disorder since long-term survival can be complicated by CHF or ATE recurrence [8,10,11,12]. Studies in cats with ATE have shown that many owners elect euthanasia [1,8,9,11].

The poor prognosis associated with feline cardiogenic ATE creates an incentive to develop improved methods for risk assessment and prevention. Several risk factors have been identified, including male sex, which is associated with an increased risk of developing HCM, and certain echocardiographic findings [7,8,11,13,14,15]. Echocardiographic findings associated with increased risk of ATE in cats include left atrial enlargement, reduced left atrial systolic function, and left ventricular diastolic and systolic dysfunction [2,3,7,8,10,11,15,16,17]. Spontaneous echogenic contrast (SEC), reduced left atrial appendage flow velocity, and identification of a thrombus within the left atrium have also been associated with increased risk of feline ATE [3,6,17,18,19,20].

If cats at risk for cardiogenic ATE can be effectively identified, prophylactic antithrombotic medications can be instituted. The decision of whether to initiate antithrombotic treatment currently relies upon echocardiography and the clinical judgment of the attending veterinarian. However, for many cats, an ATE event may be the first clinical manifestation of cardiac disease, occurring before an echocardiogram is performed. Some cats with occult cardiomyopathy may have abnormalities detected on routine auscultation that trigger a cardiac workup, but not all cats with cardiac disease have abnormal findings on auscultation [2,3,7,9,11,14,15,21]. Additionally, cats with abnormal cardiac examination findings may not undergo echocardiography due to financial constraints. Therefore, there is value in identifying diagnostic tests that could aid in identifying high-risk, non-clinical patients in a primary care setting, to help guide clinical decisions regarding further work-up and possible prophylactic treatment. Laboratory tests that could be used for risk assessment would be particularly useful given their accessibility and increased affordability.

N-terminal pro-B-type natriuretic peptide (NT-proBNP) is a cardiac biomarker commonly used to aid in the diagnosis of CHF, distinguishing cardiogenic causes of dyspnea in cats, dogs, and people [22,23,24,25]. Multiple studies have also shown that NT-proBNP can aid in identifying cats with occult cardiomyopathy versus those with normal cardiac structure; however, the low sensitivity of the point-of-care test limits its utility for screening [23,26,27,28,29]. Elevations in NT-proBNP have also been documented in association with atrial thrombi in people with mitral valve disease [30] and non-valvular atrial fibrillation [31], the latter of which has been proposed to be similar to atrial thrombi in cats with cardiomyopathy [10]. Whether similar elevations in NT-proBNP occur in cats at risk for cardiogenic ATE is unknown.

To begin assessing the relationship between NT-proBNP and feline cardiogenic ATE, this retrospective study compared NT-proBNP concentrations in cats that either had or developed ATE to cats with occult cardiomyopathy that did not develop ATE during a 1-year follow-up period. Cats with CHF but without ATE were also included in this study as a separate group for comparison, given that both ATE and CHF risk are related to left atrial enlargement. The goal was to evaluate whether NT-proBNP concentrations were different between cats that had or developed ATE compared to cats with occult cardiomyopathy that did not develop ATE within 12 months of follow-up after NT-proBNP testing. The hypothesis was that cats that had or developed ATE would have higher NT-proBNP concentrations than cats with occult cardiomyopathy.

## 2. Materials and Methods

### 2.1. Case Selection

This retrospective study enrolled client-owned cats with any form of cardiomyopathy that presented to the Cummings Veterinary Medical Center at Tufts University and had quantitative NT-proBNP testing performed. A search of the Cummings Veterinary Medical Center’s medical record database was conducted to detect cats that had NT-proBNP testing submitted to a single commercial laboratory (IDEXX Laboratories, Westbrook, ME, USA) for any reason between November 2010 and November 2016. Additional cases were identified by searching 3 other databases of cats where NT-proBNP was known to be measured: cats enrolled in previous NT-proBNP research performed by the authors in 2008–2009, cats participating in the hospital’s blood donor program from September 2011 to November 2013, and cats presenting with an ATE to the hospital between January and May of 2017.

Diagnosis of hypertrophic, restrictive, or non-specific cardiomyopathy was based on echocardiography using a standard technique performed by a board-certified veterinary cardiologist or a supervised cardiology resident [32]. Diagnosis of ATE was based on clinical signs in the affected limb(s), which could include reduced to absent arterial pulses, hypothermia, firm musculature, cyanosis of the paw pads, and paresis or paralysis [12]. The presence of CHF was determined based on clinical signs, echocardiographic findings, and radiographic findings. All cats with diagnosed or suspected non-cardiogenic dyspnea, unregulated hyperthyroidism, or systemic hypertension (systolic blood pressure > 180 mmHg) were excluded.

### 2.2. Study Design

Cats were classified into 3 groups: cats with occult cardiomyopathy (OCM group), cats with cardiomyopathy that had or developed ATE (ATE group), and cats with cardiomyopathy and CHF but no ATE (CHF group). Cats were eligible for the OCM group if they never had clinical signs of cardiac disease, including ATE, prior to the time of echocardiography and during 1 year of follow-up after NT-proBNP testing. Cats were eligible for the ATE group if they had an ATE at the time of NT-proBNP testing or if they developed an ATE within 1 year following NT-proBNP testing. Cats were eligible for the ATE group regardless of whether they had or developed concurrent CHF. Cats were eligible for the CHF group if they were in CHF at the time of NT-proBNP testing, survived at least 3 months of follow-up after NT-proBNP testing, and were not documented to ever have ATE. Cats euthanized due to worsening CHF after 3 months were still eligible for inclusion as long as they did not develop ATE prior to euthanasia. Cats were excluded from the study if they were euthanized prior to 3 months or if they died after 3 months of an unknown cause where ATE could not be ruled out.

### 2.3. Data Collection

Medical records were reviewed. Data from the echocardiogram performed closest to the date of NT-proBNP testing were recorded. In the event of missing values, measurements were performed from stored study images whenever possible. In addition to standard measurements, the presence of an intracardiac thrombus, SEC, and mitral regurgitation were recorded. Medications being administered or initiated at the time of the echocardiogram were also recorded for each cat. For the ATE group, data were recorded on whether or not antithrombotic medications had been instituted prior to the ATE event. Information on each cat’s outcome was recorded from the medical record or, if unavailable, by contacting owners and primary care veterinarians.

### 2.4. Statistical Analysis

Data distributions were examined graphically. Normally distributed data are presented as mean ± SD and skewed data are presented as median (range). Because the upper detection limit of the NT-proBNP assay was 1500 pmol/L, NT-proBNP concentrations reported as “>1500 pmol/L” were entered as “1500 pmol/L” for all statistical analyses. If the exact NT-proBNP value was available for cats in which diluted samples had been analyzed, concentrations were entered as “1501 pmol/L.” Chi-square tests were used to compare categorical data among the 3 groups (e.g., OCM vs. ATE vs. CHF groups). One-way analysis of variance tests were used to compare normally distributed data among the 3 groups with Tukey post hoc tests. Kruskal–Wallis tests were used to compare continuous variables across groups when normality assumptions were not met.

Receiver operating characteristic (ROC) analysis was used to estimate the predictive ability of NT-proBNP for feline cardiogenic ATE. The ROC curve was calculated using linear regression to model the probability of a cat being in the ATE group versus the OCM group as a function of NT-proBNP concentration. The CHF group was excluded from this analysis to control for elevations in NT-proBNP related to CHF alone, in the absence of cardiogenic ATE, although results were similar whether or not the CHF group was included. An optimal cutoff point was estimated using the Youden index (Sensitivity + Specificity − 1) [33].

Once the optimal NT-proBNP cutoff point was determined using ROC analysis, data were re-analyzed by comparing cats with an NT-proBNP concentration below the cutoff point to cats with an NT-proBNP concentration above the cutoff point. For these analyses, categorical variables were compared using Chi-square analyses and continuous variables were compared with independent t-tests (for normally distributed data) or Mann–Whitney U tests (for skewed data).

All statistical analyses were performed using commercial statistical software (Systat 13.0, SPSS, Chicago, IL, USA), with a *p*-value < 0.05 considered statistically significant for all comparisons. Bonferroni corrections were not performed despite the use of multiple comparisons given the exploratory nature of this study.

## 3. Results

### 3.1. Study Demographics

Eighty-six cats were included in this study (Table 1): 31 cats with OCM, 30 cats with CHF, and 25 cats with ATE. The most common breed was domestic short- and long-hair (OCM, *n* = 27; CHF, *n* = 25; ATE, *n* = 22). Four cats were Siamese (OCM, *n* = 1; CHF, *n* = 2; ATE, *n* = 1) and 3 cats were Maine Coon (OCM, *n* = 2; CHF, *n* = 1). There was one of each of the following breeds (study group indicated): Cornish rex (CHF), Oriental shorthair (ATE), Persian (CHF), Peterbald (OCM), and Scottish fold (ATE). There was no significant difference in breed (*p* = 0.67), age (*p* = 0.24), sex (*p* = 0.07), or underlying heart disease (*p* = 0.39) among the groups (Table 1).

Cats in the ATE group had significantly faster heart rates than cats in the CHF (*p* = 0.04) and OCM (*p* = 0.04) groups (Table 1). Cats in the ATE (*p* = 0.01) and CHF (*p* = 0.02) groups were also more likely to have a gallop sound on auscultation compared to the OCM group (Table 1). The number of cats with a cardiac murmur (*p* = 0.10), cats with an arrhythmia (*p* = 0.81), and murmur intensity (*p* = 0.39) were not significantly different among groups.

### 3.2. Echocardiography Data

Two cats in the ATE group were euthanized before a complete echocardiogram could be performed. These cats were excluded from analyses of echocardiographic data but included in the study on the basis of a thoracic point-of-care ultrasound examination performed by a cardiologist, which confirmed the presence of left atrial enlargement and cardiomyopathy.

Identification of mitral regurgitation on echocardiography was not significantly different among groups (*p* = 0.64). Cats in the CHF and ATE groups had a larger left atrium (LA) and LA to aorta (LA:Ao) ratio than cats in the OCM group (*p* < 0.001 for both comparisons; Table 2). Cats in the ATE group, but not in the CHF group, also had decreased left ventricular fractional shortening (FS; *p* = 0.01) and lower left atrial appendage (LAA) velocity (*p* = 0.02) than cats in the OCM group (Table 2). Cats in the ATE group were more likely to have a visible thrombus and SEC compared to cats in the OCM group (*p* ≤ 0.001 for all comparisons) and the CHF group (*p* = 0.02 for visible thrombus; *p* < 0.01 for SEC; Table 2). Cats in the CHF group were also more likely to have SEC than cats in the OCM group (*p* < 0.001; Table 2).

### 3.3. Treatments

As expected, medication use was more common in the CHF and ATE groups compared to the OCM group (Table 3). Of the cats receiving beta-blockers: 11 were treated with atenolol (OCM, *n* = 8; CHF, *n* = 1; ATE, *n* = 2), 4 with carvedilol (OCM, *n* = 1; CHF, *n* = 2; ATE, *n* = 1) and 2 with sotalol (both in the CHF group). Of the cats receiving an antithrombotic, 54 (95%) were treated with clopidogrel and 3 with aspirin. Only 6/25 cats (24%) in the ATE group were receiving an antithrombotic medication prior to their ATE event.

### 3.4. NT-proBNP Concentrations (All Cats)

**Figure 1:**
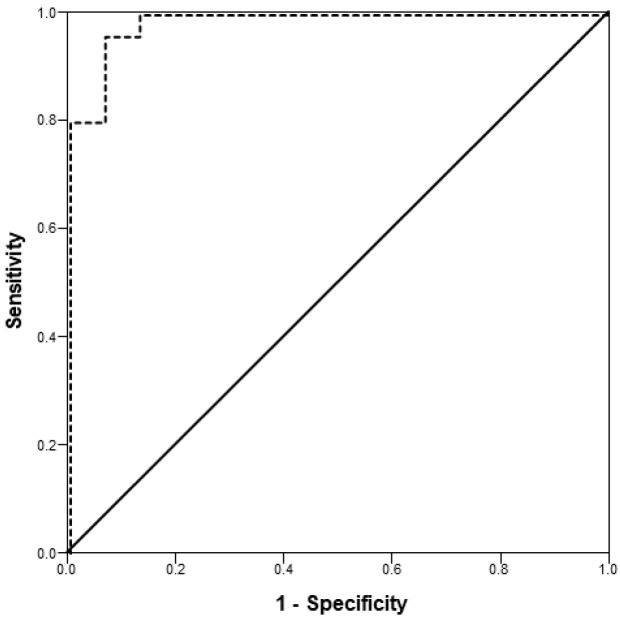
Receiver operating characteristic curve (dotted line) for modeling the probability of a cat being in the arterial thromboembolism group compared to the occult cardiomyopathy group as a function of plasma N-terminal pro-B-type natriuretic peptide (NT-proBNP) concentrations. Area under the curve was 0.985 (95% confidence interval: 0.962–1.000, *p* < 0.001). Youden’s index (J) estimated the optimal cutoff point for NT-proBNP at 491 pmol/L (sensitivity = 96.0%, specificity = 93.5%).

Cats with NT-proBNP concentrations reported as “>1500 pmol/L” (11/25 cats in the ATE group [44%] and 8/35 cats in the CHF group [23%]) were entered as “1500 pmol/L.” Cats with an exact value for NT-proBNP > 1500 pmol/L (i.e., when diluted samples were analyzed; 3 cats in the ATE group and 6 cats in the CHF group) were entered as “1501 pmol/L.” Most cats in the ATE group had NT-proBNP measured at the same visit as their diagnosis of ATE and, in some cases, CHF. The median time between measurement of NT-proBNP and the ATE event was 0 days (range, 0–256 days). Concentrations of NT-proBNP were significantly different across the 3 study groups (Table 1). Cats in the ATE (*p* < 0.001) and CHF (*p* < 0.001) groups had significantly higher NT-proBNP concentrations compared to cats in the OCM group. There was no significant difference between the ATE and CHF groups (*p* = 0.92; Table 1). There was also no significant difference between cats in the ATE group with (*n* = 19) and without (*n* = 6) CHF (*p* = 0.13). An ROC curve was generated using the NT-proBNP concentrations of the ATE and OCM groups (area under the curve: 0.985, 95% confidence interval: 0.962–1.000, *p* < 0.001). Given the output of the ROC curve, Youden’s index (J) estimated the optimal NT-proBNP cutoff point at 491 pmol/L (sensitivity = 96.0%, specificity = 93.5%; Figure 1).

### 3.5. Comparison of Cats Using NT-proBNP Cutoff

Using the optimal cutoff point of 491 pmol/L to separate ATE cats from OCM cats, cats in all 3 groups were divided into two new groups: those above the cutoff (*n* = 54) and those below the cutoff (*n* = 32; Table 4). Age (*p* = 0.39), breed (*p* = 0.47), sex (*p* = 0.07), arrhythmia (*p* = 0.24), mitral regurgitation (*p* = 0.29), and murmur (*p* = 0.92) were not significantly different between cats with NT-proBNP concentrations greater than or less than 491 pmol/L. However, cats with NT-proBNP concentrations > 491 pmol/L were significantly more likely to have a gallop (*p* = 0.03) and CHF (*p* < 0.001; Table 4). On echocardiography, cats with NT-proBNP concentrations > 491 pmol/L had a significantly larger left ventricular internal dimension in systole (LVIDs; *p* = 0.02), interventricular septal thickness in diastole (IVSd; *p* = 0.009), left ventricular wall thickness in diastole (LVWd; *p* = 0.002), maximal left ventricular wall thickness in diastole (LVWd max; *p* = 0.002), LA diameter (*p* < 0.001), and LA:Ao ratio (*p* < 0.001), but significantly lower LAA velocity (*p* = 0.02) and FS (*p* = 0.003) compared to cats with NT-proBNP concentrations <491 pmol/L (Table 4). Cats with NT-proBNP concentrations > 491 pmol/L were also more likely to have a visible thrombus (*p* = 0.01) and SEC (*p* < 0.001) on echocardiography compared to cats with NT-proBNP concentrations < 491 pmol/L (Table 4).

## 4. Discussion

As an initial step in evaluating the potential utility of NT-proBNP for assessing risk of feline cardiogenic ATE, this retrospective study compared NT-proBNP concentrations in cats with cardiomyopathy that had or developed ATE to cats that did not develop ATE. As hypothesized, cats in the ATE group had significantly higher NT-proBNP concentrations than cats in the OCM group. Furthermore, ROC curve analysis determined that NT-proBNP concentrations > 491 pmol/L differentiated cats in the ATE group from the OCM group with 96.0% sensitivity and 93.5% specificity. Concentrations of NT-proBNP > 491 pmol/L were associated with previously reported echocardiographic risk factors for feline cardiogenic ATE, including left atrial enlargement, reduced left ventricular FS, reduced LAA velocity, and SEC (Table 4) [13,16,18,19,20]. The current study also showed that NT-proBNP concentrations > 491 pmol/L were associated with an increased prevalence of SEC and intracardiac thrombi, although it should be noted that only 9 cats had a visible thrombus on echocardiography. Given the small sample size and retrospective nature of the study, these findings should be considered hypothesis-generating. Prospective studies are needed to better define this association.

The main objective of this study was to compare NT-proBNP concentrations between the OCM and ATE groups, but NT-proBNP concentrations were also evaluated in cats with CHF. Previous reports have shown that concentrations greater than 220–277 pmol/L can differentiate CHF from non-cardiogenic causes of dyspnea in cats [23,34,35]. In the current study, all cats in the CHF group had similarly elevated (>400 pmol/L) NT-proBNP concentrations. Concentrations of NT-proBNP were not different between the ATE and CHF groups. This may be related to the fact that a large percentage (76%) of cats with ATE also had CHF in this study, although there were no differences in NT-proBNP concentrations between cats in the ATE group with and without CHF.

Overall, our findings are consistent with those of a previous study that showed that NT-proBNP concentrations were positively correlated with cardiomyopathy severity [29]. Another study in cats with occult cardiomyopathy reported that NT-proBNP concentrations were positively correlated with the LA:Ao ratio and negatively correlated with FS [26]. The inability of this study to find a difference between cats with CHF and cats that developed an ATE suggests one limitation of using NT-proBNP is that it cannot differentiate between ATE and CHF risk. Instead, NT-proBNP elevation appears to generally suggest an advanced stage of cardiomyopathy that is associated with left atrial enlargement. One prior study reported that NT-proBNP was not independently associated with risk of cardiac death if left atrial size or function were accounted for using echocardiography [36].

The inability of NT-proBNP to estimate risk for a specific adverse cardiac event, specifically CHF versus ATE, may not be a clinically relevant limitation. It has previously been suggested that antithrombotic therapy is a “reasonable consideration” in all cats with HCM, dilated cardiomyopathy, and non-specific cardiomyopathy [19]. Clopidogrel is currently recommended for all cardiomyopathic cats with demonstrated risk of ATE on echocardiography, defined by significant left atrial enlargement with stage B2 disease [37]. Pending prospective studies and noting the many limitations of the current hypothesis-generating retrospective study, these results suggest that administration of clopidogrel in the absence of an echocardiogram may be reasonable in cats diagnosed with CHF or NT-proBNP concentrations > 491 pmol/L in the absence of systemic disease that may result in a confounding elevation of NT-proBNP. Notably, this cutoff is slightly lower than another study in cats with pre-clinical HCM, which suggested an NT-proBNP concentration of >700 pmol/L was associated with a four times greater risk of an adverse cardiac event [38].

In the current study, fewer than 25% of cats in the ATE group were receiving antithrombotic therapy prior to their event, which suggests a gap in prophylactic management among cats at risk for ATE. If supported by additional studies, NT-proBNP concentrations may help to address this gap by serving as an adjunct test for screening and risk assessment. Prospective studies with a larger sample size are needed to further confirm an optimal cutoff point. Further studies may also assess the utility of single versus serial measurements, and the effectiveness of NT-proBNP for screening in conjunction with other primary care diagnostics, such as thoracic radiographs or point-of-care ultrasound.

Many of the echocardiographic variables that were found to be associated with ATE in the current study are consistent with previously identified risk factors (Table 2). Cats in the ATE group had a significantly larger left atrial diameter and LA:Ao ratio compared to cats with occult disease. Left atrial enlargement has been recognized as an important risk factor for ATE and prognostic indicator in HCM [2,3,14,15,19]. In the current study, cats in the ATE group were also more likely to have SEC and intracardiac thrombi visualized on echocardiography compared to cats in the CHF and OCM groups. Measures of left-sided systolic dysfunction, such as decreased FS and reduced LAA velocity [3,20], have also been linked to ATE development and were similarly identified in the current study.

This study has many limitations that are important to consider. First, this study only included cats presenting to a single cardiology service at a veterinary teaching hospital, so our data may not reflect results from other referral centers or general practices. Additionally, as a retrospective study, not all cats had measurements performed at the same time point and long-term follow-up information was not available for all cats. Echocardiographic measurements were only recorded from a single examination date for each cat and not all cats had all echocardiographic measurements available. Many, but not all, cats were already receiving treatment at this time, which may have affected their echocardiographic measurements. There was also some variation in when echocardiography was performed in relation to NT-proBNP testing and ATE or CHF diagnosis. Standardized evaluation and monitoring in a prospective study are therefore needed before being able to make more definitive conclusions. The construction of the three groups introduces potential selection and misclassification bias which could have affected the results of the study so it would be important to confirm these results with larger, prospective studies. Given that this was a retrospective study and the commercial laboratory typically reports NT-proBNP concentrations > 1500 pmol/L as such unless dilution is requested, an important limitation is that the current results have a ceiling of 1501 pmol/L. This is an important limitation since results may have differed substantially if actual NT-proBNP concentrations > 1500 pmol/L were measured. Additional studies that measure actual NT-proBNP concentrations (using dilution, if necessary) are needed to determine optimal cutoff values. Additionally, many cats in the ATE group had NT-proBNP measured at the time of being diagnosed with a cardiogenic ATE, and, in some cases, CHF. This may have contributed to an elevation of NT-proBNP concentrations and limited the sensitivity of the proposed cutoff point. Ideally, all cats would have had NT-proBNP measured prior to onset of ATE or CHF and this would be another important consideration for future studies. Another factor that may contribute to NT-proBNP elevation is the presence of systolic anterior motion of the mitral valve [39]; this effect was documented after the completion of this study and should be considered in future investigations into the use of NT-proBNP for risk assessment. Another important limitation of this retrospective study is that some cats were already receiving medications at the time of their NT-proBNP measurement, which could have influenced not only the NT-proBNP concentration but also disease progression. The study is too small to perform multivariable analysis to determine independent effects of medications but it is important to recognize that cardiac medications could have altered the results of the study. Prospective studies with standardized medication protocols would be helpful to elucidate this issue. Another limitation is the lack of correction for multiple comparisons. This approach was elected because of the small, exploratory nature of the study but must be acknowledged for its potential to increase the risk of false-positive results and would be important to address in future, larger studies. Finally, survival times in this study were confounded by the option for euthanasia. There is no way to know if the 11 cats in the CHF group that were euthanized after 3 months but before 1 year would have developed ATE within the year and, since necropsies were not performed on all cats that died, it cannot be definitively determined that all cats that died in the CHF group did not have ATE, but they had no outward clinical evidence of ATE. Consequently, prospective studies are needed to better elucidate the potential role of NT-proBNP in evaluating risk of cardiogenic ATE in cats.

## 5. Conclusions

Identification of cats with occult cardiomyopathy that might be at risk of ATE can be challenging in primary clinical practice. Additional tools to find cats at risk of ATE would be useful, and the results of this preliminary, retrospective, hypothesis-generating study indicate that NT-proBNP is increased in cats with ATE and those at risk for ATE over a 1-year follow-up period, compared to cats with OCM. Prospective studies with matched controls are needed to better elucidate the potential predictive value of NT-proBNP in assessing risk of cardiogenic ATE in cats. If future studies confirm these findings, evaluation of quantitative NT-proBNP in asymptomatic cats may help to better identify cats that could benefit from antithrombotic medication.

## Figures and Tables

**Table 1:** Demographic and clinical characteristics of 86 cats with cardiomyopathy, categorized by main clinical presentation at the time of NT-proBNP testing: occult cardiomyopathy (OCM), congestive heart failure (CHF), or arterial thromboembolism (ATE). Data are presented as mean ± SD for normally distributed variables, median (range) for skewed data, or number of cats (% of total). The *p*-value indicates the comparison across the 3 groups, while results with different superscripts within a row indicate significant differences.

Variable	All Cats	OCM	CHF	ATE	*p*-Value
n	86	31	30	25	--
Sex: Male (castrated) Female (spayed)	66 (63) 20 (20)	25 (22) 6 (6)	25 (25) 5 (5)	16 (16) 9 (9)	0.07
Age (yrs)	8.9 ± 4.6	7.7 ± 4.8	9.4 ± 5.0	9.6 ± 3.9	0.24
Congestive heart failure	49 (57%)	0 (0%) ^a^	30 (100%) ^b^	19 (76%) ^c^	<0.001
Heart rate (per minute)	186 ± 29	180 ± 30 ^a^	180 ± 30 ^a^	199 ± 24 ^b^	0.02
Gallop	42/85 (49%)	9/31 (29%) ^a^	18/30 (60%) ^b^	15/24 (63%) ^b^	0.02
Murmur	65/86 (76%)	24/31 (77%)	19/30 (63%)	22/25 (88%)	0.10
Arrhythmia	25/85 (29%)	8/31 (26%)	10/30 (30%)	7/24 (29%)	0.81
NT-proBNP (pmol/L)	945 (19–1501)	127 ^a^ (19–913) ^a^	1484 (407–1501) ^b^	1500 (402–1501) ^b^	<0.001

Key: NT-proBNP, N-terminal pro-B-type natriuretic peptide.

**Table 2:** Echocardiographic measurements from 86 cats with cardiomyopathy, categorized as occult cardiomyopathy (OCM), congestive heart failure (CHF) or arterial thromboembolism (ATE). Data are presented as mean ± SD for normally distributed variables, median (range) for skewed data, or number of cats (% of total). For echocardiographic measurements, the total number of cats is indicated below the mean or median. The overall *p* value indicates the comparison across the 3 groups, while results with different superscripts within a row indicate significant differences.

Variable	OCM	CHF	ATE	*p*-Value
n	31	30	25	--
IVSd 2D (cm)	0.61 ± 0.12 *n* = 30	0.65 ± 0.17 *n* = 30	0.70 ± 0.15 *n* = 23	0.10
IVSd max 2D (cm)	0.67 ± 0.13 *n* = 30	0.67 ± 0.18 *n* = 30	0.74 ± 0.15 *n* = 23	0.20
LVWd 2D (cm)	0.57 ± 0.17 ^a^ *n* = 30	0.69 ± 0.19 ^a,b^ *n* = 30	0.70 ± 0.22 ^b^ *n* = 23	0.02
LVWd max 2D (cm)	0.61 ± 0.15 ^a^ *n* = 30	0.71 ± 0.19 ^a,b^ *n* = 30	0.75 ± 0.22 ^b^ *n* = 23	0.02
LVIDd 2D (cm)	1.42 ± 0.18 *n* = 30	1.52 ± 0.39 *n* = 30	1.34 ± 0.29 *n* = 23	0.09
LVIDs 2D (cm)	0.77 ± 0.12 *n* = 30	0.95 ± 0.36 *n* = 30	0.87 ± 0.32 *n* = 23	0.06
FS 2D (%)	45.2 ± 8.7 ^a^ *n* = 30	38.1 ± 12.9 ^a,b^ *n* = 30	35.5 ± 14.5 ^b^ *n* = 23	0.01
Ao 2D (cm)	0.99 ± 0.12 *n* = 31	0.92 ± 0.11 *n* = 30	0.95 ± 0.13 *n* = 23	0.07
LA 2D (cm)	1.46 ± 0.22 ^a^ *n* = 31	1.92 ± 0.39 ^b^ *n* = 30	1.87 ± 0.37 ^b^ *n* = 23	<0.001
LA:Ao ratio 2D	1.45 (1.10–2.17) ^a^ *n* = 31	2.04 (1.33–3.14) ^b^ *n* = 30	1.99 (1.16–3.36) ^b^ *n* = 23	<0.001
LAA velocity (m/s)	0.62 (0.61–0.62) ^a^ *n* = 2	0.23 (0.11–0.48) ^a,b^ *n* = 5	0.20 (0.09–0.33) ^b^ *n* = 13	0.04
Thrombus visible	0/31 (0%) ^a^	2/30 (7%) ^a^	7/23 (30%) ^b^	0.001
SEC	0/31 (0%) ^a^	10/30 (33%) ^b^	16/23 (70%) ^c^	<0.001

Key: 2D, 2-dimensional; LVIDd/s, left ventricular internal dimension in end-diastole/systole; LVWd/s, left ventricular free wall in diastole/systole; FS, left ventricular fractional shortening; IVSd, interventricular septum in diastole; LA, left atrium; Ao, aorta; LAA, left atrial appendage; SEC, spontaneous echogenic contrast.

**Table 3:** Treatment data for cats with cardiomyopathy, categorized by main clinical presentation: occult cardiomyopathy (OCM), congestive heart failure (CHF) or arterial thromboembolism (ATE). Data are presented as the number (%) of cats that were receiving each cardiac medication. Of the 86 cats in this study, 85 had treatment data recorded in their medical record. The overall *p*-value indicates the comparison across the 3 groups, while the superscripts within a row indicate how values are significantly different from another. The cat with ATE that was euthanized before treatment could be instituted has been excluded.

Drug	All Cats	OCM	CHF	ATE	*p*-Value
Furosemide	48/85 (57%)	0/31 (0%) ^a^	30/30 ^b^	18/24 (75%) ^c^	<0.001
Pimobendan	24/85 (28%)	0/31 (0%) ^a^	13/30 (43%) ^b^	11/24 (46%) ^b^	<0.001
ACE inhibitor	38/85 (45%)	5/31 (16%) ^a^	26/30 (87%) ^b^	7/24 (29%) ^a^	<0.001
Antithrombotic	57/85 (67%)	5/31 (16%) ^a^	29/30 (97%) ^b^	23/24 (96%) ^b^	<0.001
Beta-blocker	17/85 (20%)	9/31 (29%)	5/30 (17%)	3/24 (13%)	0.27
Spironolactone	2/85 (2%)	1/31 (3%)	0/30 (0%)	1/24 (4%)	0.56

Key: ACE inhibitor, angiotensin converting enzyme inhibitor.

**Table 4:** Clinical characteristics and echocardiographic data from 86 cats with cardiomyopathy, categorized by N-terminal pro-B-type natriuretic peptide (NT-proBNP) concentration above or below the receiver operating characteristic curve-derived cutoff point of 491 pmol/L. Data are presented as mean ± SD for normally distributed variables, median (range) for skewed data, or number of cats (% of total). The *p* value indicates the comparison between these two groups.

Variable	NT-proBNP < 491	NT-proBNP > 491	*p*-Value
n	32	54	--
Sex: Male (castrated) Female (spayed)	25 (22) 7 (7)	41 (41) 13 (13)	0.07
Age at NT-proBNP testing (yrs)	8.28 ± 5.11	9.21 ± 4.36	0.39
Number with CHF	3	46	<0.001
Gallop	11/32 (34%)	31/53 (59%)	0.03
Murmur	24/32 (75%)	41/54 (76%)	0.92
Arrhythmia	7/32 (22%)	18/53 (34%)	0.24
IVSd 2D (cm)	0.60 ± 0.13 *n* = 31	0.68 ± 0.15 *n* = 52	0.009
IVSd max 2D (cm)	0.65 ± 0.13 *n* = 31	0.71 ± 0.16 *n* = 52	0.07
LVWd 2D (cm)	0.57 ± 0.16 *n* = 31	0.70 ± 0.20 *n* = 52	0.002
LVWd max 2D (cm)	0.61 ± 0.15 *n* = 31	0.73 ± 0.20 *n* = 52	0.002
LVIDd 2D (cm)	1.43 ± 0.17 *n* = 31	1.44 ± 0.36 *n* = 52	0.80
LVIDs 2D (cm)	0.79 ± 0.13 *n* = 31	0.91 ± 0.35 *n* = 52	0.02
FS 2D (%)	44.6 ± 8.5 *n* = 31	37.2 ± 13.8 *n* = 52	0.003
Ao 2D (cm)	0.99 ± 0.12 *n* = 32	0.94 ± 0.12 *n* = 52	0.06
LA 2D (cm)	1.50 ± 0.29 *n* = 32	1.88 ± 0.38 *n* = 52	<0.001
LA:Ao ratio 2D	1.45 (1.10–2.56) *n* = 32	1.98 (1.16–3.36) *n* = 52	<0.001
LAA velocity (m/s)	0.62 (0.61–0.62) *n* = 2	0.21 (0.09–0.48) *n* = 18	0.02
Thrombus visualized	0/32 (0%)	9/52 (17%)	0.01
SEC	1/32 (3%)	25/52 (48%)	<0.001

Key: 2D, 2-dimensional; CHF, congestive heart failure; LVIDd/s, left ventricular internal dimension in end-diastole/systole; LVWd/s, left ventricular free wall in diastole/systole; FS, left ventricular fractional shortening; IVSd, interventricular septum in diastole; LA, left atrium; Ao, aorta; LAA, left atrial appendage; SEC, spontaneous echogenic contrast.

## Data Availability

Data available upon reasonable request.
